# Assessment of the attitude, awareness and practice of periprocedural warfarin management among health care professional in Qatar. A cross sectional survey

**DOI:** 10.1007/s11239-020-02111-w

**Published:** 2020-04-19

**Authors:** Islam Eljilany, Ahmed El-Bardissy, Arwa Nemir, Abdel-Naser Elzouki, Ihab El Madhoun, Daoud Al-Badriyeh, Hazem Elewa

**Affiliations:** 1 College of Pharmacy, QU Health, Qatar University, Doha, Qatar; 2 Department of Pharmacy, Hamad General Hospital, Hamad Medical Corporation, Doha, Qatar; 3 Department of Medicine, Hamad General Hospital, Hamad Medical Corporation, Doha, Qatar; 4 College of Medicine, Qatar University & Weill Cornell Medical College- Qatar, Doha, Qatar; 5 Department of Medicine, Al Wakra Hospital Hamad Medical Corporation, Al Wakra, Qatar; 6 Weill Cornell Medical College, Al Wakra, Qatar

**Keywords:** Survey, Warfarin, Bridging, Periprocedural management, Qatar

## Abstract

It is estimated that 10–15% of oral anticoagulant (OAC) patients, would need to hold their OAC for scheduled surgery. Especially for warfarin, this process is complex and requires multi-layer risk assessment and decisions across different specialties. Clinical guidelines deliver broad recommendations in the area of warfarin management before surgery which can lead to different trends and practices among practitioners. To evaluate the current attitude, awareness, and practice among health care providers (HCPs) on warfarin periprocedural management. A multiple-choice questionnaire was developed, containing questions on demographics and professional information and was completed by187 HCPs involved in warfarin periprocedural management. The awareness median (IQR) score was moderate [64.28% (21.43)]. The level of awareness was associated with the practitioner’s specialty and degree of education (*P* = 0.009, 0.011 respectively). Practice leans to overestimate the need for warfarin discontinuation as well as the need for bridging. Participants expressed interest in using genetic tests to guide periprocedural warfarin management [median (IQR) score (out of 10) = 7 (5)]. In conclusion, the survey presented a wide variation in the clinical practice of warfarin periprocedural management. This study highlights that HCPs in Qatar have moderate awareness. We suggest tailoring an educational campaign or courses towards the identified gaps.

## Highlights

- Warfarin periprocedural management is complex and requires multiple risk assessments and synchronized decisions.
- Clinical guidelines deliver broad recommendations in the area of warfarin management before surgery which can lead to different trends and practices among practitioners.
- This survey evaluates the current attitude, awareness, and practice among health care providers (HCPs) on warfarin periprocedural management.
- The project presented a wide variation in the clinical practice of warfarin periprocedural management and moderate awareness.

## Introduction

Oral anticoagulants (OAC) have been used for years in the treatment and prevention of thromboembolism [1, 2]. Notably, in Qatar, as well as other parts of the world, warfarin still represents a significant portion of total OAC used [3]. It has been estimated that 10–15% of OAC patients worldwide need to undergo an elective procedure on an annual basis, which may require holding OAC [4].

Periprocedural management of warfarin is a complicated process since it involves multiple steps, each of which must be assessed carefully before making a comprehensive plan. The first step is to decide whether warfarin should be interrupted. While warfarin interruption leads to decreased bleeding risk during and post-procedure, it can also increase the risk of thromboembolism [5]. Second, comes the bridging decision which may be considered to reduce the risk of thromboembolism in patients with moderate to high thromboembolic risk, however, increased risk of bleeding must be put into account [6]. In Perioperative Bridging Anticoagulation in Patients with Atrial Fibrillation trial (the BRIDGE Trial), 1884 warfarin-receiving patients with atrial fibrillation (AF) (mean CHA2DS2-VASc of 2.4) were randomly assigned to receive bridging with low molecular-weight heparin (LMWH) or a placebo-controlled bridging perioperatively [7]. The study found that bridging was associated with a more frequent incidence of major bleeding compared to non-bridging (relative risk [RR] = 0.41, 95% confidence interval [CI], 0.2–0.78, *P* = 0.005). Furthermore, LMWH did not prevent arterial thromboembolism significantly. Similarly, the Outcomes Registry for Better Informed Treatment of Atrial Fibrillation (ORBIT-AF) trial showed that the composite outcome of systemic embolism or stroke, myocardial infarction, bleeding or hospitalization was elevated in the bridging arm significantly [8]. Both studies augment the uncertainty of the need for bridging. Adding to the complexity of the bridging process is that the decision of warfarin interruption according to procedure and patient’s bleeding risks are considered another controversy. Most of the guidelines stratify the risk of thromboembolism and procedural bleeding risk into high and low, to facilitate the interruption decision [6]. Unfortunately, these classifications have some drawbacks, such as procedures with a low rate of bleeding, but with severe consequences. Categorizing these procedures as a low bleeding risk instead of a high bleeding risk procedure may be misleading. Moreover, the classification did not consider the level of intermediate bleeding risk category and did not include patients with atrial fibrillation (AF). Besides, there is a disagreement regarding the classification of some procedures such as hip/ knee replacement and prostate biopsy [9].

Collectively, it is evident that the judgment of warfarin holding and periprocedural bridging is not explicit, and decision-makers can be easily misled. This can also create several practices and attitudes among health care professionals. Consequently, a survey on the periprocedural management of warfarin was developed for a better understanding of the current practice, the gap in knowledge and attitude among health care providers in Qatar.

## Methods

### Study design and population

This study is an observational prospective cross-sectional self-administered questionnaire survey that aims to understand the practice, awareness, and attitude of health care professionals (HCPs) at Hamad Medical Corporation (HMC), Qatar, toward periprocedural management of warfarin patients.

The study was conducted over six months from July 2019 till January 2020. The participants were among physicians and clinical pharmacists from various departments involved in the periprocedural management of warfarin. A hard copy of the survey was delivered by one of the investigators. The first page of the survey contained an introductory invitation informing participants about the purpose and objectives of the survey, and confirming that the contribution to the survey was voluntary and anonymous. Convenience sampling method was used to approach the participants.

### Study setting and ethics approval

The study was performed at Al Wakra Hospital (AWH), Hamad General Hospital (HGH), and Heart Hospital (HH). These Three sites are tertiary hospitals and part of HMC, the most prominent medical institution in Qatar. Ethical approval was obtained from the Institutional Review Board (IRB) of HMC in July 2019 (Protocol# MRC-01–19-57).

### Sample size calculation

The sample size was calculated using Roasoft online calculator (www.Roasoft.com) [10], assuming that the HCPs who are involved in warfarin periprocedural management at HMC are 600. To achieve a confidence (power) level of 90% power with a 5% marginal error and taking into consideration 50% response distribution, a sample size of 187 participants was found to be adequate.

### Validation and piloting

Content and structure were checked for validity by three senior faculty members at the College of Pharmacy, Qatar University (one with expertise in pharmacy practice research, and two with cardiovascular clinical practice background). Based on their feedback, modifications were performed. A pilot version was created and disseminated to a random sample of (one internal medicine senior consultant, one cardiology specialist, one general resident physician, and one clinical pharmacist). Respondents reported that the questionnaire was well organized, clear, and with a proper sequence of questions. They also completed the survey within 15–20 min, which matched the stated duration at the invitation page of the survey.

### Survey development

The survey was designed after performing a thorough literature review using PubMed, Google Scholar, and EMBASE database in January 2019. The search focused on terms related to the HCP’s awareness and practice in warfarin periprocedural management.

The survey consisted of four domains. The first domain had 5 questions to assess the attitude of HCPs. The second domain contained 7 questions, and it evaluated the HCP`s practice. The third domain was two case scenarios with 14 questions that assessed the awareness of HCPs. The last domain collected relevant demographic and professional characteristics information of the participants. There was one question with a score ranging from 0 to 10 with one-unit intervals to rate the willingness of HCPs to recommend a genetic test to guide the duration of warfarin discontinuation. The final version of the survey consisted of 31 multiple-choice questions. Survey questions were available only in the English language.

### Measured outcome and statistical analysis

All responses were recorded in Excel document and transferred to IBM Statistical Package for Social Science (IBM SPSS 26 software; IBM, New York) for descriptive and inferential statistical analysis. Responses to demographics, professional information practice, and attitude towards periprocedural warfarin management questions, were represented as categorical variables and were expressed in frequencies and percentages. One question was presented as a continuous variable. An awareness score of one point was provided if the participant selected the correct answer for the designated question. For questions with more than one correct answer, a partial score was provided unless the participant selected all the correct answers. The overall score awareness domain was the sum of the scores of all questions under this domain. Percentage Awareness score (PAS) was calculated by dividing the total awareness score by the maximum possible score and multiplying the result by 100. Since data were non-normally distributed, Mann–Whitney U-test and Kruskal–Wallis H test were used to evaluate the effect of participants’ demographics and personal information on PAS which was expressed as median and interquartile range (IQR). A Chi-square test was performed to assess the association between different categorical values. Two-tailed *P*-value of < 0.05 was considered significant.

## Results

### Participants’ characteristics

Over six months, a total of 300 questionnaires were distributed, among which 187 questionnaires were collected (62.3% response rate). The plurality of participants (74.4%) were male, and the majority of them (69.3%) had less than 20 years of experience. Responses were received from 150 physicians (80.2%) and 37 clinical pharmacists (19.8%). Most of the physicians (31%) were specialists. A high number of participants (62.3%) were holders of a professional doctor degree such as Medical Doctorate (MD), Pharmacy Doctorate (PharmD), or equivalent degrees (Table 1).

**Table 1:** Participants’ demographics and professional characteristics

Characteristic	N (%)
Years of experience^a=3^	
0–19 years	131 (71.1)
≥ 20 years	53 (28.9)
Gender^a=3^	
Male	137 (74.4)
Female	47 (25.6)
Highest degree received^a=4^	
Bachelor’s degree	29 (15.9)
Academic degree	40 (21.8)
Professional doctor degree (MD, Pharm D)	114 (62.3)
Healthcare provider	
Clinical pharmacist	37 (19.8)
Physician	150 (80.2)
Physician ranking	
Resident	37 (19.8)
Specialist	58 (31.0)
Consultant	50 (26.7)
Senior consultant	5 (2.7)
Physician specialty^a=1^	
Internal medicine	52 (34.7)
Cardiology	20 (13.5)
Anesthesiology & Surgery	56 (37.7)
Other	21 (14.1)

^a^Missing response. Other, family medicine, geriatric medicine, general medicine

### Awareness of periprocedural warfarin management

The overall median (IQR) of PAS was moderate 64.28% (21.43). Out of 14 awareness questions, the major deficiency was identified in 5 questions [less than 50% of responders chose the right answer(s)]. Firstly, there is the awareness of the type of surgeries that do not require warfarin interruption (right response rate = 26.2%). Also, there is the awareness regarding the time at which patients must stop warfarin and stop LMWH prior to surgery (right response rate = 42.2%, 47.1%, respectively). Furthermore, bridging decision was another obstacle in both case scenarios (right response rate = 38% & 47.6%). In bridging decision scenarios, we found apparent contrast in response among specialties. While 15% of cardiologists agreed on continuing warfarin for patients undergoing cataract or tooth extraction procedure, only 5% of anesthesia and surgery physicians preferred not to stop warfarin (Table 2).

**Table 2:** Survey domains, questions and responses

Attitude Domain	Respondents (%)
1. How do you perceive warfarin interruption during periprocedural management based on your clinical experience?	
A. Underused	32 (17.1)
B. Used appropriately	77 (41.2)
C. Overused	47 (25.1)
D. Do not know	31 (16.6)
2. How do you perceive heparin bridging use during warfarin interruption in the periprocedural management based on your clinical experience?	
A. Underused	34 (18.2)
B. Used appropriately	82 (43.9
C. Overused	50 (26.7)
D. Do not know	21 (11.2)
3. How do you perceive the risk of bleeding when considering bridging with heparin during warfarin periprocedural management?^a=3^	
A. Not important	14 (7.6)
B. Somewhat important	52 (28.3)
C. Very important	109 (59.2)
D. Do not know	9 (4.9)
4. How do you perceive the patient burden and cost when considering bridging with heparin during warfarin periprocedural management?^a=2^	
A. Not important	44 (23.8)
B. Somewhat important	61 (33.0)
C. Very important	66 (35.7)
D. Do not know	14 (7.5)
5. If there is a genetic test which informs you more accurately about the optimal duration of warfarin interruption before surgery, on a scale of 0–10, how much do you recommend the patient to do this genetic test?	187 (100)

Practice domain	
1. On average, how often do you provide care for patients requiring warfarin periprocedural management?^a=5^	
A. 1–2 patients/week	160 (87.9)
B. 3–5 patients/week	13 (7.1)
C. 6–8 patients/week	6 (3.3)
D. More than 8 patients/week	3 (1.7)
2. Who is typically responsible for warfarin management during the periprocedural period in your unit?^b^	
A. Clinician performing the surgery or procedure	70 (37.6)
B. Anticoagulation clinic	60 (32.2)
C. A clinician who prescribed warfarin	71 (38.2)
D. Other	31 (16.6)
3. Which guidelines do you follow for warfarin periprocedural management?^b^	
A. American College of Chest Physician (ACCP)	42 (22.5)
B. American College of Cardiology (ACC)	64 (34.2)
C. American Society of Hematology [11]	11 (5.9)
D. National Institute for Health and Care Excellence (NICE)	22 (11.9)
E. European Society of Cardiology [12]	26 (13.9)
F. Clinical Excellence Commission (CEC)	0 (0.0)
G. HMC’s guideline	94 (50.3)
H. Other ^c^	10 (5.3)
4. How often do you encounter canceling or postponing a procedure due to elevated INR around the procedure time despite warfarin interruption?^a=3^	
A. Never (0%)	12 (6.5)
B. Rarely (1–25%)	67 (36.4)
C. Sometimes (26–75%)	72 (39.1)
D. Frequently (76–99%)	15 (8.2)
E. Always (100%)	5 (2.8)
F. Don’t know	13 (7.0)
5.1 How often do you encounter a situation (No warfarin interruption is needed before an elective procedure) for patients requiring periprocedural management of warfarin?^a^	
A. 0–25%	149 (85.2)
B. 26–50%	14 (8.0)
C. 51–75%	10 (5.7)
D. 76–100%	2 (1.1)
5.2 How often do you encounter situation (Warfarin interruption is needed before the procedure but WITHOUT heparin bridging) for patients requiring periprocedural management of warfarin?^a=13^	
A. 0–25%	72 (41.5)
B. 26–50%	70 (40.2)
C. 51–75%	28 (16.0)
D. 76–100%	4 (2.3)
5.3 How often do you encounter situation (Warfarin interruption is needed before the procedure but WITH heparin bridging) for patients requiring periprocedural management of warfarin?^a=11^	
A. 0–25%	33 (18.8)
B. 26–50%	41 (23.3)
C. 51–75%	63 (35.8)
D. 76–100	39 (22.1)
6. Would you check the patient’s INR on the day before or the day of the procedure?^a=3^	
A. For all the patients	153 (83.2)
B. Only for patients who DID NOT have warfarin interrupted before the procedure	6 (3.3)
C. Only for patients who HAD warfarin interrupted before the procedure	15 (8.1)
D. No need to check for the INR before the procedure	1 (0.5)
E. Do not know	9 (4.9)
7. On which of the below scales, do you assess this patient’s stroke risk?^a^^=^^11^	
A. CHA_2_DS_2_-VAS	125 (67.2)
B. CHADS_2_ score	32 (17.2)
C. Other	1 (0.5)
D. Do not know	28 (15.1)

Awareness domain	
1. Which is the most considerable factor to you during warfarin periprocedural management?^a^^=^^3^	
A. Type of surgery	7 (3.8)
B. Patient`s risk of bleeding	6 (3.3)
C. Bleeding risk of the procedure	6 (3.3)
D. Risk of thrombosis	1 (0.6)
E. All the above	162 (89.5)
F. Other	2 (1.1)
2. In which of the following procedures/surgeries would you decide to continue warfarin during the procedure time?^b^	
A. Tooth extraction	49 (26.2)
B. Resection of abdominal aortic aneurysm	10 (5.3)
C. Cataract	67 (35.8)
D. Cholecystectomy	10 (5.3)
E. None of the above	84 (44.9)
F. Other	8 (4.3)
Case scenario 1: A 55-year-old female patient currently on warfarin for deep vein thrombosis (DVT) that occurred 10 years ago. Her INR has been within the range lately (most recent INR reading is 2.3) and all her other labs are unremarkable. Patient has also hypertension and hypothyroidism. Patient will have a colonoscopy with possible polypectomy in 10 days	
3. Would you stop warfarin prior to the scheduled colonoscopy?	
A. Yes	146 (78.1)
B. No	28 (15.0)
C. Do not know	13 (7.0)
4. If the patient has to stop warfarin, when do you advise the patient to stop it before the surgery?	
A. > − 7 days of the surgery	8 (4.3)
B. − 7 to − 5 days of the surgery	79 (42.2)
C. − 4 to − 3 days of the surgery	77 (41.2)
D. − 2 to − 1 days of the surgery	20 (10.7)
E. Do not know	3 (1.6)
5. Would you bridge this patient with heparin?^a^^=^^2^	
A. Yes	88 (47.6)
B. No	89 (48.1)
C. Do not know	8 (4.3)
6. Considering that the patient will be bridged with low molecular weight heparin (LMWH), when do you start LMWH before the surgery?^a^^=^^8^	
A. − 5 days of the surgery	41 (22.9)
B. − 4 days of the surgery	6 (3.3)
C. − 3 days of the surgery	47 (26.3)
D. − 2 days of the surgery	39 (21.8)
E. − 1 day of the surgery	34 (19.0)
F. Do not know	12 (6.7)
7. When do you stop LMWH before the surgery?	
A. − 2 days of the surgery	8 (4.3)
B. − 1 day of the surgery	88 (47.1)
C. On the day of the surgery	87 (46.5)
D. Do not know	4 (2.1)
8. What is the safe INR limit for doing the surgery?^a^^=^^2^	
A. ≤ 1.2	16 (8.6)
B. ≤ 1.5	146 (79.0)
C. ≤ 2	18 (9.7)
D. Do not know	5 (2.7)
9. If the patient has to stop warfarin, when do you resume it considering no bleeding post-operatively?^a^^=^^3^	
A. The night of or the day following the surgery	110 (59.5)
B. + 2 to + 3 days of the surgery	61 (33.4)
C. + 4 to + 5 days of the surgery	1 (0.5)
D. > + 5 days of the surgery	3 (1.7)
E. Do not know	9 (4.9)
10. When do you check INR after restarting warfarin?^a^^=^^6^	
A. + 1 to + 2 days	65 (35.5)
B. + 3 to + 5 days	102 (55.7)
C. + 5 to + 7 days	10 (5.5)
D. > + 7 days	2 (1.1)
E. Do not know	4 (2.2)
Case scenario 2: A 75-year-old male patient currently on warfarin for atrial fibrillation. His INR has been within the range lately (most recent INR reading is 2.5) and all his other labs are unremarkable. Patient will have a hip replacement planned in 10 days	
11. Would you stop warfarin prior to the scheduled hip-replacement?^a^^=^^2^	
A. Yes	166 (89.7)
B. No	4 (2.2)
C. Do not know	15 (8.1)
12. If the patient’s atrial fibrillation is non-valvular and he has a history of controlled hypertension, diabetes, and gout, would you decide to bridge him before the surgery?^a^^=^^2^	
A. Yes	97 (52.4)
B. No	71 (38.4)
C. Do not know	17 (9.2)
13. If you knew that this patient had a history of mechanical mitral valve replacement, would you decide to bridge him before the surgery?	
A. Yes	175 (93.6)
B. No	3 (1.6)
C. Do not know	9 (4.8)
14. If you knew that this patient had non-valvular atrial fibrillation and history of cardioembolic stroke 2 months ago, would you decide to bridge him before the procedure?	
A. Yes	150 (80.2)
B. No	19 (10.2
C. Do not know	18 (9.6)

^a^Missing response

^b^Choose all that apply. INR, International Normalization Ratio

^c^Other as identified by responders: American College of Anesthesia, American College of Gastroenterology, American College of Surgeons, American Society of Gastroenterology

**Fig. 1:**
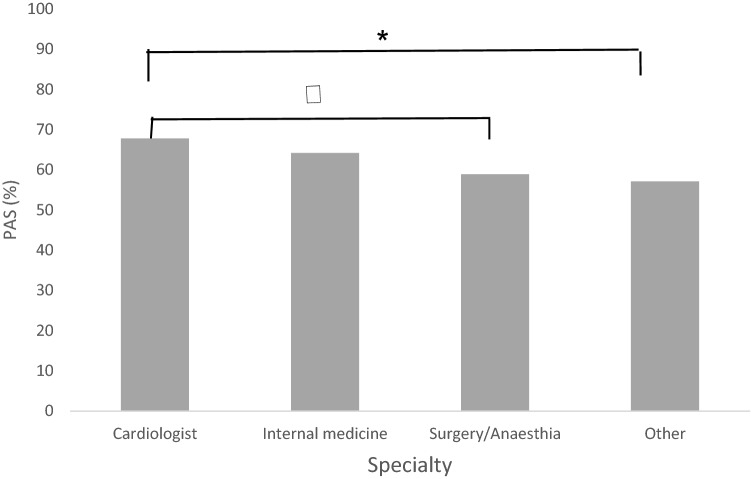
Median PAS across physicians’ specialties. Bars represent median percentage of awareness score (PAS) across physicians’ specialties. Statistical significance was tested using the Kruskal–Wallis test (*P* < 0.05) followed by post-hoc pairwise comparison. Results are expressed as median (IQR) PAS. PAS, Percentage Awareness Score. *P-value = 0.004;^†^*P*-value = 0.036

In terms of the effect of demographics and professional information on the participants’ awareness, the following were the most significant findings. Participants holding master’s or professional degree achieved significantly better median (IQR) PAS, than participants holding a Ph.D. degree [60.71% (18.75), 64.28% (16.07) vs. 50% (17.86), *P* = 0.004, *P* = 0.007 respectively]. Pharmacists showed a significantly superior median (IQR) PAS compared to physicians [75% (20.54) vs. 60.71% (20.54), *P* = 0.001]. As expected, when cardiologists were compared to surgery/anesthesia physicians and other specialties, they attained a significantly higher median (IQR) PAS score [67.85% (24.11) vs. 58.92% (20.98, *P* = 0.036), 57.14% (37.95, *P* = 0.004) respectively]. Similarly, internalists got significantly superior median (IQR) PAS score versus other specialties [64.28% (17.86) vs. 57.14% (37.95), *P* = 0.007] (Fig. 1). Table 3 shows the effect of baseline and professional characteristics on PAS.

**Table 3:** Effect of baseline and professional characteristics on percentage awareness score

Variable	Median PAS (IQR)	*P*-Value*
Years of experience		0.74
0–19 years	60.71% (19.64)	
≥ 20 years	64.28% (22.32)	
Gender		0.49
Male	60.71% (25)	
Female	64.28% (16.07)	
Highest degree received		0.011
Bachelor’s degree	57.14% (25.57)	0.126^a^
Master’s degree	60.71% (18.75)	0.335^a^
Professional doctor degree (MD, Pharm D)	64.28% (16.07)	
Doctorate degree	50% (17.86)	0.007^a^
Current position		0.001
Clinical pharmacist	75% (21.43)	
Physician	60.71% (20.54)	
Physician Ranking		0.02
Resident	57.14% (28.57)	0.141^b^
Specialist	58.92% (19.64)	0.861^b^
Consultant & Senior consultant	64.28% (17.86)	
Physician specialty		0.009^*^
Internal medicine	64.28% (17.86)	0.437^c^
Cardiology	67.85% (24.11)	
Anesthesiology & Surgery	58.92% (20.98)	0.036^c^
Other	57.14% (37.95)	0.004^c^

*PAS* percentage awareness score

^*^P value < 0.05 was tested using the Kruskal–Wallis test for the comparison of PAS between the following factors (highest degree, current position, and main specialty), while Mann–Whitney U test was used for the comparison of PAS between following factors (years of experience & gender)

^a^Post-hoc pairwise comparisons of bachelor’s, master’s and doctorate degree vs. professional doctor degree (MD, Pharm D)

^b^Post-hoc pairwise comparisons of residents’ and specialists’ Vs. consultants & senior consultants

^c^Post-hoc pairwise comparisons of anesthesiology/surgery physicians and other specialties vs cardiology

### The practice of HCPs in periprocedural warfarin management

Most of the respondents (87.9%) reported that they deal with 1–2 warfarin patients per week undergoing a procedure. There was a statistically significant association between specialty and who is accounted for the direct management of these cases (*P* < 0.001). Half of the cardiologists (50%) indicated that the anticoagulant clinic is responsible for making plans for the patient, while a similar proportion of internal medicine agreed on warfarin prescriber as the main responsible party. In contrast, 37.5% of surgeon and anesthesia physicians declared that clinician performing the procedure is liable to handle these cases.

About a third of the HCP indicated that they encounter a reschedule/ cancellation of the procedure due to elevation in INR some or most of the time.

**Fig. 2:**
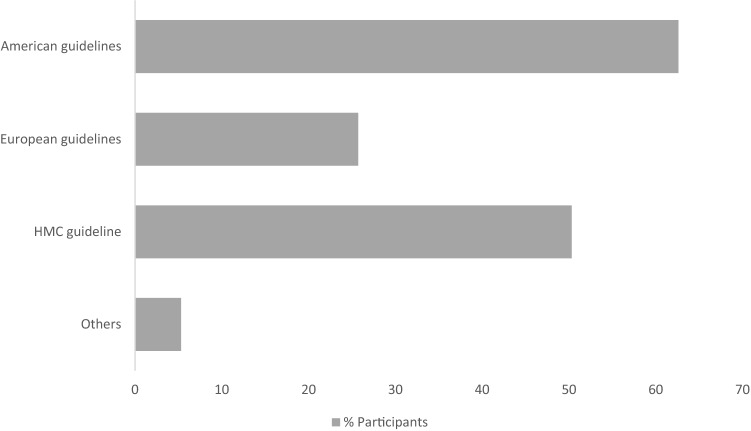
Participants use of the different guidelines in warfarin periprocedural management. Bars represent percentage of participants and the guidelines followed in warfarin periprocedural management. Other as identified by responders: American College of Anesthesia, American College of Gastroenterology, American College of Surgeons, American Society of Gastroenterology, Clinical Excellence Commission

American guidelines were the most widely used for guidance (62.8%) followed by HMC’s guidelines (50.3%), and then the European guidelines (25.8%) (Fig. 2).

In terms of warfarin interruption, 85.2% of respondents indicated that around 75% of patients need warfarin discontinuation before elective surgery, and that about half of those patients (56.1%) will require bridging.

When the respondents were asked to indicate which criteria are used to assess patient’s stroke risk, just under 70% reported that they use the CHA2DS2-VASc score, while fewer (17%) reported the use of CHADS_2_ score.

### Attitude towards periprocedural warfarin management

A chi-square test for association was conducted between demographics and warfarin periprocedural management attitude. Females significantly perceived more than males that warfarin interruption, and heparin bridging are overused (34% vs 22.6%, *P* = 0.003, *P* = 0.034 respectively). More emphasis on the difference in the attitude of physicians and pharmacists; whereby, more physicians believed that the cost of bridging is very important (38.5%vs 24.3%, *P* = 0.042). Participants expressed a good level of interest in using genetic tests to guide periprocedural warfarin management [median (IQR) score (out of 10) = 7 (5)].

## Discussion

In this study, we attempted to assess the attitude, knowledge, and practice of HCPs in Qatar on periprocedural management of warfarin patients undergoing a procedure. The main finding of the study was that participants’ awareness is moderate. In a recent study in Qatar, a similar level of awareness was achieved among HCPs on direct oral anticoagulants (DOACs) [13]. Three areas of knowledge deficiency were the driver of the decline in awareness level in the current study. Firstly, conflicting ability to determine the duration of discontinuation of warfarin prior to the procedure. This is surprising given the fact that a clear recommendation in the 2017 American College of Cardiology (ACC) guideline states that, warfarin should be held 5–7 days before an elective procedure [9]. A second area of deficiency was the inconsistencies between HCPs on who bridge warfarin patient and the duration of preoperative parenteral anticoagulation when a decision to bridge is made. Thirdly, the majority of participants were lacking awareness of the type of procedures that do not require warfarin interruption, such as cataract and tooth extraction due to their low-risk of bleeding [14, 15]. Whether the low score achieved in these elements is due to true lack of awareness or judgement from clinical practice and experience is hard to assess. Regardless, we believe that applying inappropriate timing, duration of warfarin interruption or bridging can yield significant risk of bleeding and thromboembolic events. It was also found that cardiologists were the best in continuing warfarin in procedures with low risk of bleeding, while most of the surgeons still stopped warfarin. This is potentially due to the cardiologists’ attention to patient’s thromboembolic risk, while surgeons give more attention to the procedure’s bleeding risk. Results from a survey that evaluated the practice patterns in the Unites States for bridging AC showed that 25% and 45% decided not to interrupt warfarin during dental extraction and cataract surgery, respectively [16]. Bridge or Continue Coumadin for Device Surgery Randomized Controlled trial (BRUISE CONTROL) has shown that maintaining warfarin with an INR of ≤ 3 on the day of the procedure in patients undergoing implantation of pacemakers or cardioverter defibrillators was associated with significantly less bleeding than warfarin discontinuation along with bridging with heparin (Odds ratio:0.19; p < 0.001) [17].

Another critical observation in the survey is that clinical pharmacists had better awareness scores compared to physicians. A possible explanation for this might be that clinical pharmacists have a reasonable knowledge of pharmacokinetics and pharmacology of warfarin, and are frequently involved with warfarin dosing and periprocedural management through anticoagulation clinics and in-patient services [12]. A significant difference was also noted among the physician’s specialties, where cardiologists and internalists achieved the highest scores. This result is likely related to these specialties being more involved in the management of warfarin patients.

As expected, HCPs holding professional degrees had a superior awareness than fresh graduate HCPs holding a bachelor’s degree. Surprisingly, HCPs with PhD got a lower awareness score than HCPs with a professional degree. It is possible that practical training plays a significant factor in determining the awareness level. We also observed that the position or rank was positively associates with the awareness of periprocedural warfarin management (highest in consultants/senior consultants). While one may expect from recent graduates to have better awareness, extensive clinical practice appears to have a vital role in augmenting awareness levels. These results are also in alignment with the previous survey on DOACs awareness in Qatar [13].

Response to the involvement in periprocedural warfarin management was another interesting finding. The majority of each specialty were biased towards their own practice. For instance, cardiologists, being the specialty running jointly or in close relation to the anticoagulation clinics in Qatar agreed on the anticoagulant service as the main responsible party for periprocedural management. Similarly, surgeons and anesthesiologists referred to the clinician performing the procedure as the responsible, while internalist referred to the warfarin prescriber as the responsible party. These findings are consistent with data from a recent survey in which respondents distributed the responsibility among cardiologists, surgeons, internalists and anticoagulant services to manage warfarin periprocedural (56%, 36%, 28%,and 27%, respectively) [16].

In addition to our main findings above, respondents revealed that warfarin is discontinued in the majority of patients who will undergo elective surgery. This was reflected when most of the participants chose to stop warfarin in cataract and tooth extraction surgeries in separate questions. Similar trends were expressed by participants in this survey and those described by Starks et al. [18], Krahan et al. [19], and Balbino et al. [20] (75%, 83%, and 83% interrupted warfarin preoperatively correspondingly). We believe that this clinical practice leans towards fear of bleeding events from warfarin much more than thromboembolic events. However, HCPs in our study stated that almost half of those patients undergoing warfarin discontinuation would require bridging to protect them from thromboembolic events. Both of these practice behaviors (exaggerated discontinuation and bridging) may put the patients at higher risk of thromboembolism and bleeding, respectively. This comes also against the recent expert call to reduce the use of bridging during preoperative management due to the increased risk of bleeding from heparin use [21]. In this report, it was estimated that over 90% of patients receiving warfarin therapy should not receive bridging anticoagulation during periprocedural management. This conclusion was based on accumulating evidence that rated overall and major bleeding significantly higher in bridged rather than non-bridged patients by 2–5 folds while there was no difference in the risk of thromboembolism between both arms [22].

As an area of future research and possible clinical translation we asked HCPs on their opinion to use a genetic test as a tool to help in personalizing the duration of warfarin interruption before surgery. Remarkably, the survey articulated the interest of HCPs (especially pharmacists) in recommending this tool to their patient in the future. These results are in agreement with Elewa et al. [23] findings in 2015, which showed that pharmacists had more willingness and positive attitude towards the application of pharmacogenetics in practice when compared to physicians in Qatar.

A key strength of the current survey is that it investigated different domains (attitude, knowledge, and practice) of various specialties involved in warfarin periprocedural management. On the other hand, this study had some limitations. First, there is a potential for sampling bias since we surveyed a governmental hospital only, i.e. HMC, which could affect the generalizability of the results. Despite a high response rate in this survey (62.3%), some HCPs did not agree to participate possibly due to lack of knowledge or interest which may have had an impact on the generalizability of the results. To overcome that, we intentionally used a paper-based survey instead of an online version to increase the response rate. In addition to the above limitations, survey fatigue, and lack of required time to answer the survey are obstacles that could have affected the response quality. We tried to solve this issue by limiting the number of case scenarios. Moreover, validation of the questionnaire helped to ensure it had appropriate time and clarity. Lastly, and similar to other survey-based studies, our findings may be distinct from what applies in practice.

In conclusion, this research highlights that HCPs in Qatar have moderate awareness of warfarin periprocedural management with a lack of standardized practice. Practice leans to overestimate the need for warfarin discontinuation due to fear of bleeding risk. Besides, it overestimates the need for bridging to overcome thromboembolic risk. Additionally, HCPs are interested in applying pharmacogenetics to their practice to gage the duration of warfarin discontinuation. Future work should focus on reassessing practitioners’ knowledge after providing well-designed education campaigns.
